# Rank Aggregation Methods and Tools in Genomic Data Analysis

**DOI:** 10.2174/0113892029320249240830060611

**Published:** 2024-09-11

**Authors:** Wenping Zou, Savannah Mwesigwa, Sayed-Rzgar Hosseini, Zhongming Zhao

**Affiliations:** 1 Center for Precision Health, McWilliams School of Biomedical Informatics, The University of Texas Health Science Center at Houston, Houston, TX 77030, USA

**Keywords:** Rank aggregation, meta-analysis, data integration, bayesian, stochastic, genomics

## Abstract

Rank aggregation (RA) is the process of consolidating disparate rankings into a single unified ranking. It holds immense potential in the field of genomics. RA has applications in diverse research areas, such as gene expression analysis, meta-analysis, gene prioritization, and biomarker discovery. However, there are many challenges in the application of the RA approach to biological data, such as dealing with heterogeneous data sources, rankings of mixed quality, and evaluating the consolidated rankings. In this review, we present an overview of the existing RA methods with an emphasis on those that have been tailored to the complexities of genomics research. These encompass a broad range of approaches, from distributional and heuristic methods to Bayesian and stochastic optimization algorithms. By examining these techniques, we aim to equip researchers with the background knowledge needed to navigate the intricacies of RA in genomics data integration effectively. We review the practical applications to highlight the relevance and impact of RA methods in advancing genomics research. As the field continues to evolve, we identify open problems and suggest future directions to enhance the effectiveness of rank aggregation in genomics, by addressing the challenges related to data heterogeneity, single-cell omics and spatial transcriptomics data, and the development of clear and consistent evaluation methods. In summary, RA stands as a powerful tool in genomics research, which can offer deeper insights and more comprehensive data integration solutions.

## 1 INTRODUCTION

Rank aggregation (RA) is a statistical and computational methodology that consolidates ranked lists from multiple sources, and is used in different fields such as marketing, voting, web page ranking, and psychology [1-3]. Recently, RA methods have been increasingly utilized to combine high-throughput datasets for the identification of consensus genetic signatures [4-6]. Genomics research typically involves the analysis of various types of data, such as gene expression profiles, genetic variants, and protein interactions, which are often collected from different sources and under different experimental conditions. Considering the diversity, complexity, and noisy characteristics in biological data, it is critical to establish a consensus to interpret the findings more accurately. The main goal of RA in genomics is to extract common patterns from diverse genomic datasets, ultimately forming an integrated consensual list that improves the reliability and interpretability of the genomics data [7-9]. Furthermore, by prioritizing consistently high-ranked genes, proteins, or genetic variants from various sources, a consensual rank helps simplify the data interpretation and uncover potentially significant insights and discoveries, driving progress in understanding genomics and its implications for health, disease, and biological systems.

RA methods have shownseveral unique features, which can explain their widespread popularity [10-13]. First, they help reduce dimension, while enabling the detection of subtle genomic patterns. Second, they help reduce the impact of individual study biases, leading to more robust and reliable results. Finally, RA methods facilitate reconstructing more accurate biological networks and identifying a more robust list of enriched pathways. Thus, they have been widely utilized, for example, to develop meta-analysis methods in genomics that combine consensus information to improve statistical meaning [14, 15]. They also help identify consensus gene signatures or pathways across different studies to facilitate the extraction of meaningful biological markers or targets. Particularly, when integrating diverse genomics data and identifying consensus rankings, RA methods provide a clearer view of complex biological phenomena [16, 17]. Furthermore, in drug discovery, RA methods have been used to prioritize potential drug targets or compounds by combining data from various assays and high-throughput screens [18-20]. Importantly, RA methods can bridge the gap between basic genomics research and clinical applications by consolidating evidence for the utility of specific biomarkers or treatment strategies [6, 11].

In this review article, we aim to highlight the ever-growing importance of RA methods in genomics by providing an overview of RA method categories and their practical applications. Our objective is to provide an update for researchers in genomics, bioinformatics, and computational biology, by offering insights into the rapidly evolving field of RA, and its potential relevance in harmonizing and interpreting diverse genomic datasets. Our ultimate goal is to contribute to the data-driven discoveries and advancements in genomics research by examining the strengths and limitations of various RA techniques, and also exploring emerging trends and opportunities.

## 2 RANK AGGREGATION: THE BASIC CONCEPT

In this section, we first briefly introduce the basic concept of RA, which provides the foundation for our subsequent discussions. Omics technologies produce high-dimensional data, which can be represented as ranked lists reflecting the relative quantities of interest among a list of components, such as genetic variants, genes, or proteins. The resulting ranked lists may vary widely depending on the sources of data, methodologies and experimental conditions. In such cases, RA methods are essential to construct a unified, robust, and more accurate ranking that encompasses all these conditions. Fig. (**1**) illustrates the concept of RA by considering a list (*L*) including n genes *L* = {g_1_, g_2_, g_3_, …, g_n-1_, g_n_}, and by denoting their relative ranking as *Γ* = {*L*_p_ ≥ *L*_q_ ≥ ⋯ ≥ *L*_z_}, where the symbol “≥” indicates ranking superiority. For instance, in *Γ*_1_, g_1_ is ranked higher than g_2_, and in *Γ*_2_, g_3_ surpasses g_2_. Depending on the data source and experimental conditions, a set of multiple distinct ranked lists is created, which is represented as {*Γ*_1_, *Γ*_2_, *Γ*_3_, …, *Γ*_i-1_, *Γ*_i_}. The goal of RA is to find an aggregated ranking list, denoted as *Γ*_∗_, which more accurately represents the underlying truth than each individual *Γ*_i_.

Typically, RA methods aggregate complete lists that provide precise ranks for all items of interest, but this is rarely applicable in genomics, because certain genes of interest are often absent from individual lists due to a lack of laboratory measurements or removal during preprocessing and quality control steps, resulting in partial lists. Additionally, individual lists may sometimes provide exact rankings for only the top genes, creating a top-ranked list. Thus, a variety of computational RA methods using different strategies have emerged to tackle these challenges.

## 3 CLASSIFICATION OF RANK AGGREGATION METHODS

We adopt a classification scheme, as proposed by Lin *et al.* in 2010 [6], which divides the RA methods into four categories: 1) Distributional, 2) Heuristic, 3) Bayesian, and 4) Stochastic optimization approaches. As shown in Fig. (**2**), this classification summarizes the main features of these diverse strategies employed in the RA methods.

### 3.1 Heuristic-based Approaches

Heuristic approaches use a practical method, which is not guaranteed to be optimal, and are not based on exhaustive computations, but are still sufficient for reaching an approximate solution. They tend to prioritize simplicity and often rely on intuitive methods. In this section, we will explore two common types of heuristic RA algorithms, namely Borda's approaches and Markov Chain-based ones.

#### 3.1.1 Borda’s Approaches

Borda-inspired methods are popular for their simplicity and ease of use. Borda's original method determined aggregate ranks by computing the arithmetic average of full-ranked lists. Over time, various aggregation functions and modifications have been proposed and implemented, which have expanded their usefulness to top-k lists. These functions utilize basic statistical measures, including the arithmetic mean, median, geometric mean, and L2-norm of the rankings from individual sources to combine rank data. These methods are illustrated as Mean, Median, Geometric mean, and L2-norm (Table **1**).

#### 3.1.2 Markov Chain Models

Markov chain models are popularly used in RA methods because of their ability to capture state transitions that represent the ranking variations among items. Markov chain models in RA are focused on pairwise ranking information, which indicates how one item is ranked relative to another in the same list. This information is valuable in constructing transitions between different states of the Markov chain. Accordingly, the state space of the Markov chain is defined by all the items to be ranked. A transition matrix is then constructed, which describes the probabilities of moving from one state (item) to another based on pairwise rankings. Ultimately, the goal is to create an ergodic Markov chain that is irreducible and aperiodic, and also allows for movement between any pair of states, and does not get stuck in loops. The stationary distribution of this Markov chain is crucial as it represents the long-term probabilities of the system found in each state. In the context of RA, this stationary distribution reflects the aggregate rankings of the items. It is important to note that stationary distribution assigns higher probabilities to states, which are higher ranked. Therefore, by constructing the Markov chain appropriately, we ensure that the stationary distribution effectively determines the aggregate rankings of the items. The transition probabilities are designed based on the intuition that higher-ranked items should have a higher likelihood of being reached from other items. Markov chain methods offer flexibility in assigning transition probabilities based on the researcher's objectives. Different algorithms can be employed, providing a range of approaches to leverage the available pairwise ranking information. In summary, Markov chains for RA involve modeling the process of transitioning between different ranked states, allowing for a comprehensive understanding of how items move and settle in their final aggregate ranking positions. Various methods have been available for assigning these probabilities, depending on specific objectives. To formulate the transition matrix, four Markov chain-based approaches have been proposed: MC1, MC2, MC3, and MC4 [5, 6, 21]. MC1: The current state-page P is set. Then, the next state, which is ranked higher than (or equal to) P is picked uniformly from the multiset of the whole pages by the same method, that is, from the multi-set: U_𝑖_{Q **|** ꚍ_𝑖_(Q) ≤ ꚍ_𝑖_(P)}. MC2: If the current state is page P, then the next state is chosen by first picking a ranking ꚍ uniformly from all the partial lists ꚍ_1_, …, ꚍ_k_ containing P, then picking a page Q uniformly from the set: {Q│ꚍ(Q) ≤ ꚍ(P)}. This chain considers the fact that we have several lists of rankings, not just a collection of pairwise comparisons among the pages. MC3: If the current state is page P, then the next state is chosen as follows: we first pick a ranking ꚍ uniformly from all the partial lists ꚍ_1_, …, ꚍ_k_ containing P, then uniformly pick a page Q that is ranked by ꚍ. If ꚍ(Q) ≤ ꚍ(P), the state moves to Q. MC4: If the current state is page P, then the next state is chosen as follows: we first pick a page Q uniformly from the whole ranked union. Then, a majority of the lists ꚍ ranks both Q and P. If ꚍ(Q) ≤ ꚍ(P), the state moves to Q.

### 3.2 Distribution-based Approaches

A method falls under the category of distribution-based if it relies on a probabilistic latent model or utilizes distributional information of any statistic derived from the rank data. Developed by Thurstone in the 1920s, Thurstone's model, also known as Thurstone's scaling, is an early exemplar of distribution-based RA methodologies [22, 23]. However, due to the necessity of numerous base rankers for parameter estimation, it is not applicable in genomic studies, and thus a distribution-based RA method was developed by Stuart *et al.* to identify the conserved co-expression patterns in different organisms [24]. Their approach involved using a distribution-based RA technique to identify metagenes (*i.e*., sets of genes across multiple organisms, whose protein sequences are one another's best reciprocal BLAST hit) that are co-expressed across diverse experiments and multiple species. They used a diverse dataset from DNA microarray experiments encompassing humans, worms, flies, and yeast to perform their analysis. This dataset also reflected various conditions, including developmental stages, stress, and disease. The method first ranked meta-gene pairs (*m, m’*) based on Pearson correlation coefficients and then using order statistics, generated corrected *P*-values (Q statistic) from a joint cumulative distribution of an n-dimensional order statistic (equation 1 and 2, *s*: organism; *k*: rank ratios for the meta-gene pair; *n*: number of the organisms). This Q statistic quantitatively measures the likelihood of observing specific ranking configurations by chance, and could then be used as a final score (aggregated rank) to select significant metagene pairs.


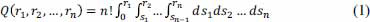


The following recursive formula was composed to compute the above integral, where r_0_=0 and the recursive call to Q supplies all of the original arguments except the *(k-i+1)^th^* argument.


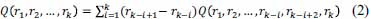


Aerts *et al.* proposed a more efficient formula than Stuart's, which can handle moderate to large data sources (N), and hence significantly reduce computational complexity [25]. Additionally, Aerts *et al.* observed that the non-uniform distribution of Q statistics under the null hypothesis limited their direct application as p-values. To address this, they used a fitting approach where a distribution was fitted for each possible number of ranks. This distribution was used to approximate p-values. For *N ≤ 5*, Q statistics for randomly and uniformly drawn rank ratios approximate a beta distribution, while for *N > 5*, they can be modeled by a gamma distribution.

Classical RA methods aim to achieve the highest coherence possible; however, this may not always be the best approach for aggregating preferences, mainly when some rank lists are unreliable. In 2012, Kolde *et al*. proposed Robust Rank Aggregation (RRA) to tackle this issue [13]. RRA distinguishes reliability at the gene level by using a null model based on randomly ordered gene lists, similar to a permutation test in the absence of additional information. The method utilizes a scoring approach for rank vectors to identify consistently high-ranked genes across multiple preference lists. It addresses potential non-informative studies and formulates the probability of observing a rank statistic under a null model using binomial or beta distribution. The resulting minimum *P*-value, denoted as ρ(r), is used to order rank vectors and assess statistical significance through correction for multiple hypothesis testing, where the Bonferroni correction is recommended for computational efficiency. Wald *et al.* conducted a 2012 investigation comparing Mean Aggregation with the RRA method. Surprisingly, their findings suggested that Mean Aggregation not only rivaled RRA, but in many instances surpassed it [26]. This surprising finding underscores the potential of Borda’s methods such as Mean Aggregation as an uncomplicated yet highly effective approach, positioning it as a compelling choice across numerous applications. At present, however, the most commonly used method in genomics is RRA, which is known for its computational efficiency and its flexibility to handle datasets of various sizes [13, 27-29].

### 3.3 Stochastic Optimization Approaches

Stochastic optimization-based approaches are widely used in bioinformatics to minimize the disagreement between the input lists and the aggregate rankings. The optimization criterion applied to Cross-Entropy Monte Carlo (CEMC) conforms to the generalized Kemeny guideline, which is distance-dependent and would use either Kendall's tau or Spearman’s footrule distance [5]. Kendall's tau counts the number of pairwise discordances between the two lists, and Spearman's footrule distance is basically the sum of the absolute differences between the two lists over all elements. The Cross-Entropy Monte Carlo (CEMC)-based RA has been proposed to efficiently explore the space of potential aggregate lists [12].

Suppose Q_A_(*k*) is the ranked list with *k* elements (for example, genes), from algorithm A. For ease of the illustrated method, all of the input lists are cut off to the same size. Let *Q_1_, . . ., Q_L_* be L *top-k* lists with respect to the same space but different underlying ranking mechanisms ꚍ_1_, . . ., ꚍ_L_. Assign Q = 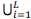 Q_𝑖_, which is the union of the elements from the lists. The unarranged n elements are indexed from *1 to n*. To find the estimate of P, an ordered subset of Q, we pursue the formula as follows:


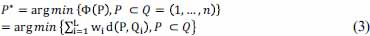


where w_i_ is a weight vector and *d* is a distance measurement.

CEMC algorithm may result in multiple algorithms when performing the task. Therefore, the Order Explicit Algorithm (OEA) was introduced. In OEA, the orders of the elements in the optimal lists are clearly specified in the probability matrix [12]. Suppose *X = (X_ij_)_n×m_* is a random matrix, where each item X is variable (taking values 0 or 1). The matrix has the constraints that each column sums to 1, and each row sums to at most 1. Suppose *v = (p_ij_)n×m* is a probability matrix, where each column sums to 1. Then each column variable *X_j_ = (X_1j_, X_2j_, ..., X_nj_)* follows a multinomial distribution with a sample size of 1 and the probability vector *v_j_ = (p_1j_, . . ., p_nj_)*. These variables are subject to the aforementioned constraints on the joint column variables.

### 3.4 Bayesian Approaches

Recognizing that some rankers may provide more informative results than others, the incorporation of weighting factors into the base rankers becomes essential. Deng *et al.* introduced the Bayesian Aggregation of Rank Data (BARD) method, which has demonstrated superior performance, especially when applied in conjunction with diverse base rankers [30]. BARD uses a Bayesian method to reformulate the order-based RA issue into a Bayesian model selection one. The ranking problems would be reformulated *via* BARD as follows: we assume that relevant entities U_r_ and noisy background entities U_b_ are two subsets in the set U without overlapping. To distinguish the relevant entities U_r_ from the background U_b_, we set the group indicator *I_i_* (i∈U), in which the rankings ꚍ_1_, . . ., ꚍ_i_ are mutually independent in a given indicator vector *I* (*I_i_* = 1, if i ∈ U_r_; or 0, if i ∈ U_b_). Since the consideration of relevant entities and background ones for ꚍ_i_, the “relevant” group serves as a good measure to rank the entity. BARD associates a quality parameter to each base ranker to quantify their reliability. It assigns entities into irrelevant *versus* relevant categories and assumes that the relative ranking of the irrelevant items is random, while those of the relevant entities are assumed to follow a power-law distribution. Ultimately, the aggregate ranker is obtained based on the posterior probability of each item being relevant. Adopting a similar strategy, the Bayesian Iterative Robust Rank Aggregation (BIRRA) method was introduced by Badgeley *et al.* in 2015 [31]. It uses the mean rank as the initial criterion for distinguishing between the irrelevant and relevant entities.

## 4 APPLICATIONS OF RANK AGGREGATION METHODS IN GENOMICS

In this section, the applications of each RA method are detailed in the specific algorithms, and features are summarized in Table **1**. Further, the statistical counts of each method’s appearance in the gene meta-analysis are listed in Table **2**. The comparison of applications indicates the features of each method and provides useful instructions on selecting specific RA methods in specific analysis.

### 4.1 The Applications of Heuristic-based Approaches

In 2006, a rank-aggregation approach was proposed for integrating results from multiple microarray studies, addressing issues of incomparability in expression levels. By employing meta-search algorithms inspired by computer science literature, particularly those utilizing Markov chain frameworks, the study demonstrated the effectiveness of the approach in aggregating results from prostate cancer microarray studies [32]. Later, in 2012, Sengupta *et al.* discussed the established utility of RA in bioinformatics, highlighting the need for weighted aggregation scenarios and introducing a modified Markov chain approach for prioritizing sources of ranking, which outperformed other contemporary techniques in gene ordering from microarray analysis and microRNA target prediction [33]. In 2020, Kolmykov *et al.* focused on creating integrated datasets of transcription factor binding sites (TFBSs) using a three-stage application of RA, revealing a high saturation of site motifs in the most reliable TFBSs and their preference for open chromatin regions [34]. Deshpande *et al.*, (2022) presented a novel algorithm, Single-cell Inference of Networks using Granger Ensembles (SINGE), a method for inferring gene regulatory networks from single-cell gene expression data. SINGE uses kernel-based Granger causality to accommodate the irregular distribution of pseudotimes, which represent the cells' progression through biological processes [36]. The core innovation was in employing a modified Borda aggregation technique to merge predictions from various models. This technique ranks potential gene interactions by compiling consensus rankings from multiple analyses, enhancing the robustness of the inferred networks against the complexities of single-cell data. Jiang *et al.*, 2017 focused on enhancing gene microarray data classification through an improved RA method [37]. It employed wavelet decomposition for data processing and a variety of algorithms for feature selection. The novel aspect was its advanced Borda count method for RA, which assigns weights to rankings from different algorithms, improving the accuracy and reliability of cancer gene data classification by considering the distinct reliability of each selection method.

To address the challenge of feature selection in high-dimensional SNP datasets, Kavakiotis *et al.*, 2016 formulated feature selection as a ranking aggregation problem, drawing from principles of social choice and voting theory, to effectively evaluate and select SNP features for various medical and biological applications. These studies collectively underscored the versatility and efficacy of RA methods in tackling complex biological and computational problems, offering insights and solutions that advanced scientific understanding and practical applications [38].

### 4.2 The Applications of Distribution-based Approaches

In the section “Exploring Genomics Applications of RA in the Literature,” we observe that the Robust Rank Aggregation (RRA) method emerges as the most frequently employed approach for RA in genomic studies. RRA is a distribution-based method and here we exemplify its application in investigating its applications in genomics across various diseases. In head and neck squamous cell carcinoma (HNSCC), RRA integration of multiple microarray datasets led to the identification of *AURKA*, *BIRC5*, and *UBE2C* as potential prognostic biomarkers [27]. Similarly, in papillary thyroid carcinoma (PTC), RRA facilitated the discovery of diagnostic and prognostic hub genes such as *ABCA6*, *ACACB*, *RMDN1*, and *TFPI* [28]. Additionally, in prostate cancer (PCa), RRA integration of microarray data unveiled *LMNB1*, *TK1*, *ZWINT*, and *RACGAP1* as candidate biomarkers associated with Pca clinical features [35]. Lastly, for dilated cardiomyopathy (DCM), RRA aided in identifying 117 significant DEGs, with subsequent analyses revealing a 7-gene signature predictive of DCM, including *ANKRD1*, *COL1A1*, *MYH6*, *PERELP*, *PRKACA*, *CDKN1A*, and *OMD* [29]. Finally, Spooner *et al.* used ensemble feature selection with data-driven thresholds, notably Robust Rank Aggregation and threshold algorithm from information retrieval, to enhance the stability of Alzheimer’s disease biomarker discovery, achieving up to 34% improvement in stability over individual selectors, and reflecting current findings in AD literature [36]. These findings collectively underscore the efficacy of the distribution-based methods, in particular, RRA in integrating diverse datasets to elucidate disease mechanisms and identify potential diagnostic and therapeutic targets across various pathological conditions.

### 4.3 The Applications of Stochastic Optimization Approaches

In 2001, Sese and Morishita introduced an RA method for gene expression profiles, employing a ranking system based on statistical properties, notably the Poisson distribution, to order genes by significance. This method, originally applied to web search results by Dwork *et al.*, was adapted for biological databases, using the Kendall tau distance to calculate the optimal RA, thereby facilitating the comparison and synthesis of independent experimental data [37]. In 2007, Pihur, *et al.* presented a weighted RA method using a cross-entropy Monte Carlo approach by incorporating Kendall tau distance and Spearman’s footrule distance to evaluate clustering algorithms in gene expression data effectively. This methodology has facilitated a comparison of algorithms, enhancing the selection process with its adaptability and precision [38].

### 4.4 The Applications of Bayesian Approaches

Bayesian methods are still in their infancy and are still not commonly used in Genomics compared to Robust Rank Aggregation, a distribution-based method as explained in the section “Exploring Genomics Applications of Rank Aggregation in the Literature”. Nevertheless, in 2018, Li *et al.*, introduced the Bayesian latent variable approach (BiG) for RA by focusing on the aggregation of partial and top-ranked lists in genomic studies. This method attempted to address the limitations of existing approaches by incorporating the effects of clustering and accommodating partial and top-ranked lists. Their simulation studies demonstrate BiG’s superior performance over other popular RA methods (MEAN, MED, GEO, L2, RRA, BARD, BIRRA, Stuart, CEMCk, CEMCs, MC1, MC2, and MC3) under various practical settings. Additionally, its application to non-small-cell lung cancer data illustrated the approach’s utility, emphasizing its effectiveness in genomic research contexts where traditional methods may fall short [39].

In 2022, Wang *et al.* presented a systematic comparison of ranking aggregation methods for gene lists, utilizing both simulated and real data, including datasets on SARS-CoV-2, cancer (non-small cell lung cancer), and bacteria (macrophage apoptosis) [16]. The authors introduced a novel simulated data generation method to better reflect real-world heterogeneity and to evaluate various ranking aggregation methods, highlighting Meta-Analysis by Information Content (MAIC) as particularly effective across scenarios with varying data heterogeneity and noise levels. The results were synthesized in a practical flowchart for selecting an appropriate ranking aggregation method based on specific dataset characteristics, offering a valuable resource for meta-analysis in genomic research.

### 4.5 The Statistics of Genomics Applications of Rank Aggregation in the Literature

Our examination of the PubMed research papers related to “Rank Aggregation” during the last twenty years indicates a significant rise in the use and interest of this approach. The “Robust Rank Aggregation” algorithm has gained considerable attention over other methods, such as Bayesian techniques, particularly in gene analysis studies, as shown in Fig. (**3**). We also conducted a bibliometric analysis of research articles on 'Rank Aggregation' published between 2010 and 2023. Using VOSviewer v1.6.20, we created a comprehensive bibliometric map that includes 115 scientific terms from 292 references. The map provides a visual representation of the complex web of knowledge and highlights the changing trends in research over time. In the early publications, the focus was mainly on 'Methodology and Performance Analysis.' During this phase, researchers were primarily concerned with developing and improving the methodologies related to RA techniques. This period saw the emergence of innovative “rank aggregation methods” [11, 13, 31]. RA was used in diverse domains, such as data mining, to extract insights from fMRI data and examine biomedical research networks [40, 41]. RA methods were also used to enhance techniques like association rule mining, clustering, active learning, annotation diagnosis, bandit algorithms, and integrated bioinformatics analysis [42, 43].

In recent years, there has been a shift towards 'Multi-omics' research, particularly in clinical applications highlighting the dynamic nature of research in this domain. Advanced multi-omics approaches now take the center stage, and offer a fresh perspective on clinical prognosis and survival analysis for various diseases, such as bipolar disorder, major depressive disorder [44], Alzheimer's disease [45, 46], autoimmune thyroid disease [47], diabetic nephropathy [48], non-alcoholic steatohepatitis [49], hepatocellular carcinoma [28, 50, 51], bladder cancer [52], and castration-resistant prostate cancer [53]. These studies underscore the increased versatility of RA in data integration, knowledge discovery, and bioinformatics research. Furthermore, they imply that RA is a powerful and versatile technique, which is relevant across diverse scientific disciplines. Thus, its adaptability can contribute significantly to knowledge domains and facilitate transformative advances in various research fields.

## 5 THE CHALLENGES AND OPPORTUNITIES

To produce more robust rankings for specific items, researchers may need to utilize a wide variety of algorithms and computational tools. The core concept of RA involves amalgamating diverse “base rankers” *via* multiple ranking functions, and in genomics, this can facilitate extracting meaningful insights from diverse data. However, a major challenge is that in real-world scenarios, there are frequently disparities in the quality provided by individual base rankers, and thus a need to distinguish high-quality from lower quality base rankers. However, this challenge also presents an opportunity for innovation. Newer methods that offer the ability to assign pre-determined weights to the base rankers, allow for a more refined treatment of rankers based on their quality and enhance the overall reliability and utility of genomics RA techniques. In the early stages, the straightforward Borda's approaches provided a solid foundation for aggregating rank data. However, as the complexity of genomics RA has grown, more sophisticated options, such as Markov chain approaches, have emerged. Markov chain methods introduce a more detailed process and can deal with scenarios involving partial lists, top-k ordering, or uneven comparisons. Of note, Markov chain techniques can produce different outcomes by varying the required assumptions in certain circumstances. This paves the way for approaches that are more specific on analysis tasks. Conversely, optimization-based methods emphasize well-defined optimization criteria, which typically quantify the disagreements among the input lists and the generated aggregate rankings. For example, the Kemeny guidelines serve as a framework for establishing a consensus ranking, while minimizing discrepancies among the input lists [54]; and this involves the assignment of weights to the distances between the aggregate rankings. The method of choice for measuring these distances affects the quality of determination of the aggregate list, and this aspect highlights the challenge and opportunity inherent in optimization methods. Also, the selection of distance measurement techniques has been shown to significantly impact the effectiveness and reliability of genomics rank aggregation outcomes [55, 56].

In the field of genomics, rank aggregation has witnessed the emergence of different approaches tailored to address the differences in ranking quality. These methods include Robust Rank Aggregation (RRA), Bayesian aggregation of Rank Data (BARD) built on Bayesian principles, Bayesian iterative RRA (BIRRA), and Bayesian Aggregation in Genomic applications (BiG). Each of these methods offers a distinct framework for tackling the complexities of genomics RA, with the potential to offer unique solutions to the challenges at hand. One crucial example of the essential role of RA methods in genomics and transcriptomics research is highlighted in the study by Wang *et al.* [16]. The research provided valuable insights into selecting appropriate aggregation methods for datasets that are characterized by varying levels of “heterogeneity in quality, noise levels, and a mix of ranked and unranked data”. According to their findings, 'Meta-Analysis by Information Content' (MAIC) outperformed other methods in large datasets [57], including those containing both ranked and unranked lists, such as SARS-CoV-2 data. MAIC demonstrated exceptional performance across various evaluation metrics. Another method, rMixGEO (geometric mean), which employs simple statistical approaches, also exhibited commendable performance and matched MAIC in scenarios with low heterogeneity. Furthermore, their study emphasized the significant impact of heterogeneity and mean noise on the performance of methods like BIRRA and BiGbottom (Bayesian approaches), which produce better results than MAIC for small simulated datasets characterized by high heterogeneity and low mean noise [16]. However, they are less robust, when dealing with a large mean noise level. This variation in performance across mixed datasets highlights the importance of method selection tailored to specific data characteristics, such as the mix of ranked and unranked sources, heterogeneity in data quality, and noise. The authors provide a practical flowchart to assist researchers in selecting appropriate methods based on dataset attributes.

Due to the technological advancement, transcriptomic data have been generated by various technologies, such as microarray, RNA-seq, and single-cell RNA-seq. To apply RA for a specific study, it is crucial to process the data by considering factors such as technology platforms, batches, and coverage of the transcriptomics (*e.g*., number of genes with measurable expression and expression level). The batch effect can be commonly corrected by methods such as ComBat and surrogate variable analysis (SVA) for bulk-RNA data and Harmony for single-cell data. The normalization process can be conducted by quantile normalization, log transformation, and variance stabilization. It is crucial to evaluate the difference in sequence depth and coverage of the transcriptomic data before the RA analysis and to filter those low-coverage datasets.

There has been an increased utilization of RA methods across diverse fields of biological research, such as mRNA expression, microRNA targets, ChIP-seq, and transcription factor binding sites [34, 58]. However, it is worth noting that despite these applications, RA has seen limited use in the context of protein datasets and drug discovery, suggesting untapped potential in these areas [18]. The increase in multi-omics data highlights the utility of RA in downstream genomic data analysis. In response to this growing need, researchers are actively developing scalable and parallelized aggregation methods, aiming to handle the escalating volume of data efficiently [59]. Additionally, the increased availability of genome catalogs, such as the GWAS (genome-wide association studies) Catalog (https://www.ebi.ac.uk/gwas/) and PGS (polygenic score) Catalog (https://www.pgscatalog.org/), presents a promising avenue for applying RA techniques to efficiently retrieve relevant variants, genes and associated traits across diverse studies. Furthermore, there is a need in the research community to establish benchmark datasets and standardize evaluation metrics specifically for RA methods. This standardization initiative will enhance the rigor of comparisons among various approaches, and facilitate the identification of the most suitable method for specific genomics research applications.

Single-cell omics has been under rapid growth, providing high-resolution insights into cellular heterogeneity. Rank aggregation methods can be effectively applied to single-cell transcriptomics and spatial transcriptomics to integrate and analyze data from multiple cells, conditions, or spatial locations. Briefly, after gene expression data processing (including data missingness and mitochondria gene expression filtering [60]), normalization (including spatial smoothing for spatial transcriptomics data), and batch effect correction [61], we can aggregate rankings of gene expression with each cell cluster or cell type to find consistent genes, pathways, and transcriptional programs [62]. The consensus ranking can be also performed by different conditions, time points, cell states, and locations of tissue sites using methods such as Robust Rank Aggregation, Borda count, *etc*. Specifically for spatial transcriptomics data, ranking genes can be performed within each spatial spot or similar spots within each tissue region. Furthermore, gene ranking in RA method development can be benefited from the foundation models trained by millions of cells across human organs and tissues, such as scGPT [63], and generative AI based approaches, such as GENEVIC [64]. In summary, by applying RA methods, researchers can integrate complex single-cell and spatial transcriptomics data, enhancing the understanding of cellular heterogeneity, tissue organization, and biological processes.

Another promising avenue of exploration involves the integration of additional biological metadata, such as splicing information and structural data, into the RA process. This integration may hold the potential to enhance the biological relevance of aggregated rankings, ultimately leading to more insightful and contextually meaningful outcomes. Collectively, these ongoing endeavors within genomics, are geared towards providing profound insights and comprehensive data integration solutions, which will empower researchers with the tools needed to unlock the full potential of multi-omics data.

## CONCLUSION

As genomics research advances rapidly, RA methods demonstrate their great utility as important tools for extracting reliable biological information by integrating diverse datasets. In this paper, we explore the foundational concepts of RA, its historical development, and various methods that are applied to real-world data. There is an increased need for task-specific RA techniques tailored to the unique challenges of genomics data integration. The discrepancies in the reliability of individual rankers in real-world scenarios also require the development of methods that allocate predefined weights to base rankers and facilitate a nuanced treatment based on their quality. We have discussed early-stage aggregation methods, including basic Borda’s statistics, and more advanced techniques, such as Markov chain and optimization methods. Each of these approaches offers unique advantages and considerations. The application of RA methods has been witnessed in diverse areas of biological research, ranging from mRNA expression and microRNA targets to single-cell expression and transcription factor binding sites. As the volume of multi-omics data expands exponentially, its significance in downstream genomic data analysis is vital. There is a need in the research community for benchmark datasets and standardizing evaluation metrics to enable rigorous comparisons among the different approaches. Furthermore, integrating additional biological metadata, such as recently emerged spatial transcriptomic and traditional molecular structural data, can enhance the biological relevance of aggregated rankings. Considering these advances, it is evident that RA methods have become necessary tools in the genomics researcher's toolkit, and are poised to catalyze progress in genomics research and offer meaningful insights and comprehensive solutions in our quest to decipher the intricacies of biology.

## Figures and Tables

**Fig. (1):**
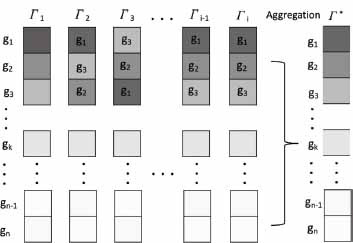
Illustration of the process of rank aggregation. Each column Γ represents a distinct method to generate a ranked list of genes 'g.' The ranked lists represented as Γ_1_ to Γᵢ are combined into a unified list, Γ∗, which reflects the consensus rank. The color gradient in the figure corresponds with the ranking, where darker shades depict higher ranks, providing a visual cue to easily identify the relative significance of each gene within the aggregated list.

**Fig. (2):**
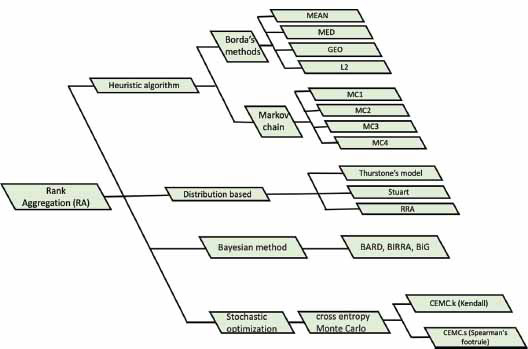
Overview of the classification scheme for RA methods. This diagram presents the categorization of RA methods into four distinct approaches: 1) Distributional, 2) Heuristic, 3) Bayesian, and 4) Stochastic optimization approaches.

**Fig. (3):**
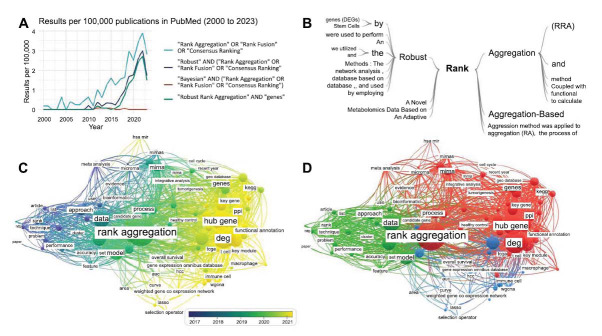
A global view of the rank aggregation (RA) applications in literature. (**A**) Trends in RA methods: This line chart depicts the evolving landscape of RA methods. It showcases the increasing adoption of rank aggregation (light blue) and the growing prominence of Robust Rank Aggregation (dark blue) compared to Bayesian approaches (red), with a specific focus on genetic studies(green). The line plot presents normalized publication counts per 100,000 annually, sourced from PubMed (https://esperr.github.io/pubmed-by-year/). (**B**) Word Tree Analysis of “Rank Aggregation” in PubMed. A Word Tree Analysis of the PubMed literature on “Rank Aggregation” underscores the prevalence of “robust” as a significant prefix (https://esperr.github.io/pub-trees/). (**C, D**) The Bibliometric Map of Scientific Terms in PubMed Publications (Jan 2010 - Feb 2023) on 'Rank Aggregation': This map, created using VOSviewer (v1.6.20), visualizes the co-occurrence of terms in titles and abstracts. Each term's proximity indicates co-occurrence and its size reflects frequency. The map includes 115 terms from 292 references. (**C**) The map overlaid with publication dates highlights evolving research trends over time. (**D**) Terms are grouped into three clusters: 'Methodology and Performance Analysis' (green), 'Multi-omics' (red), and 'Clinical Prognosis and Survival Analysis.'

**Table 1:** Description of commonly suggested aggregation functions.

**Method**	**Description**	**Equation**
Mean	Calculates the average by summing values and dividing by the count, which is a widely used measure of central tendency. Suitable for datasets without extreme values, providing a balance of all values in the calculation.	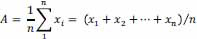
Median	Calculates the median of a set of values, and hence is a robust measure that is less sensitive to outliers. It is particularly useful for avoiding skewing effects from extreme values.	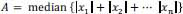
Geometric mean	It is suitable for scenarios, where proportional relationships are of interest, providing a measure of central tendency that considers the multiplicative nature of data.	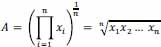
L2 norm	It allows flexibility in adjusting sensitivity to individual values through the choice of the power parameter (p). The mean (arithmetic average) is a special case when p = 1.	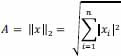

**Table 2:** An overview of RA methods implemented in genomic analysis. The Table outlines the various characteristics of these methods, including their names, corresponding URLs for further exploration, underlying algorithms, distinctive features, and relevant references.

**Method**	**URL**	**Algorithm**	**Feature**	**References**
BARD	https://www.jstor.org/stable/24247433	Bayesian	Robust; works better with variable equality of base rankers.	[30]
BIRRA	https://doi.org/10.1093/bioinformatics/btu518	Bayesian	Robust to noise; External and internal statistics are not required; Ranked lists required only.	[31]
BiG	https://doi.org/10.1002/sim.7920	Bayesian	Accommodates more complex situations; dealS with partial and top-ranked lists.	[39]
RRA	(Aerts *et al.*, 2006).org/10.1093/bioinformatics/btr709	Distribution-based	Easy & efficient to compute and robust; significant score; non-informative lists	[13]
CEMC.k: CEMC based on Kendall CEMC.s: CEMC based Spearman's footrule distance	https://link.springer.com/book/10.1007/978-1-4757-4321-0	Stochastic optimization	Weighted lists assigned; addresses the unequal base rankers of interest.	[32]
GEO, L2, MEAN, MED	https://doi.org/10.1093/bib/bbx101	Borda's count	Empirical, simple, and efficient.	[11]
MC1, MC2, MC3	https://doi.org/10.1002/wics.111	Markov Chain	Construct transition matrix.	[6]
Stuart	https://www.science.org/doi/10.1126/science.1087447	Distribution-based	Fast and interactive gene prioritization	[24]

## LIST OF ABBREVIATIONS

BARD: Bayesian Aggregation of Rank Data
BIRRA: Bayesian Iterative Robust Rank Aggregation
CEMC: Cross-Entropy Monte Carlo
DCM: Dilated Cardiomyopathy
HNSCC: Head and Neck Squamous Cell Carcinoma
MAIC: Meta-Analysis by Information Content
OEA: Order Explicit Algorithm
PCa: Prostate Cancer
PTC: Papillary Thyroid Carcinoma
RA: Rank Aggregation
RRA: Robust Rank Aggregation
SINGE: Single-cell Inference of Networks using Granger Ensembles
SVA: Surrogate Variable Analysis
TFBSs: Transcription Factor Binding Sites
